# Effects of Non-invasive Vagus Nerve Stimulation on Inflammatory Markers in COVID-19 Patients: A Systematic Review and Meta-Analysis of Randomized Controlled Trials

**DOI:** 10.7759/cureus.70613

**Published:** 2024-10-01

**Authors:** Amira Mohamed Taha, Amr Elrosasy, Ahmed S Mohamed, Ahmed Elmorsy Mohamed, Abdallah Bani-Salameh, Abdelmonem Siddiq, Shirin Cadri, Ahmed Elshahat, Atef A Abdelmonteser, Moaz E Abouelmagd

**Affiliations:** 1 Neurology, Faculty of Medicine, Fayoum University, Fayoum, EGY; 2 Neurology, Medical Research Group of Egypt (MRGE), Arlington, USA; 3 Ophthalmology, Faculty of Medicine, Cairo University, Cairo, EGY; 4 Internal Medicine, Faculty of Medicine, Merit University, Mansoura, EGY; 5 Neurology, Faculty of Medicine, Tanta University, Tanta, EGY; 6 Medicine, Jordan University of Science and Technology, Irbid, JOR; 7 Therapeutics, Faculty of Pharmacy, Mansoura University, Mansoura, EGY; 8 Internal Medicine, Universitatea de Medicină și Farmacie "Grigore T. Popa" Iași, Iași, ROU; 9 Internal Medicine, Al-Azhar University, Cairo, EGY; 10 Medicine and Surgery, Al-Azhar University, Cairo, EGY; 11 Neurology, Faculty of Medicine, Cairo University, Cairo, EGY

**Keywords:** covid-19, inflammation, nvns, systemic inflammatory markers, vagus nerve stimulator

## Abstract

The international healthcare community has encountered several difficulties because of the COVID-19 pandemic brought on by SARS-CoV-2. COVID-19 can lead to an abnormal immune response that features excessive inflammation, so targeting the vagus nerve through non-invasive vagus nerve stimulation (nVNS) may hold promise as an intervention. This meta-analysis aimed to examine the outcomes of using nVNS on different inflammatory biomarkers in COVID-19 patients. Up until May 2023, we performed a review of online databases. We included randomized controlled trials (RCTs) that discussed how nVNS affected patients with COVID-19's clinical outcomes. Using the Revman 5.4 software (Cochrane, London, United Kingdom), a meta-analysis was carried out to find the pooled mean difference (MD), with 95% confidence intervals (CIs), of nVNS effects on different inflammatory biomarkers, including interleukin-10 (IL-10), C-reactive protein (CRP), IL-6, and cortisol levels.

The review included four RCTs involving 180 COVID-19 patients. Following nVNS treatment, there was a significant increase in IL-10 levels (MD = 1.53, 95% CI: 0.77, 2.29; p < 0.001). CRP levels (MD = -2.24, 95% CI: -4.52, 0.05; p = 0.06), IL-6 levels (MD = 4.07, 95% CI: -3.16, 11.32; p = 0.27), cortisol levels (MD = 1.45, 95% CI: -11.67, 14.57; p = 0.83), and D-dimer levels (MD = -0.47, 95% CI: -1.31, 0.38; p = 0.28) did not differ significantly. These findings suggest that nVNS may positively impact certain inflammatory markers in COVID-19 patients, suggesting that nVNS could be a beneficial adjunctive treatment.

## Introduction and background

Introduction

A reported death rate of over six million people has been associated with the global emergence of COVID-19, which has affected 767,726,861 people worldwide [1]. Despite advances in medical research over the past couple of years, the treatment options for COVID-19 are still limited, and the rates of death remain concerning. One of the key pathological features of COVID-19 infection is inflammation. Studies have shown that severe COVID-19 infection is associated with the excessive release of cytokines, including interleukin (IL)-2, IL-6, IL-10, and tumor necrosis factor-α (TNF-α). Acute respiratory distress syndrome (ARDS), septic shock, acute kidney failure, acute lung injury, and central nervous system impairment were all reported to be affected by these biomarkers [2-5].

Since inflammation plays a crucial role in developing COVID-19, a good therapeutic approach would be to modulate this heightened immune response. A novel anti-inflammatory method, non-invasive vagus nerve stimulation (nVNS), has recently been tested in multiple conditions. Elderly patients who undergo total joint arthroplasty usually suffer from delayed neurologic recovery, but it has been reported that nVNS helps reduce this delay by suppressing inflammatory biomarkers and cytokines [6,7].

The cholinergic anti-inflammatory pathway (CAP) is activated by stimulation of the auricular vagus nerve. This triggers vagus nerve signals to the central nervous system, subsequently initiating a cascade of events that suppress the release of inflammatory cytokines. This sequential procedure is thought to be involved in the control of the immune response [8]. Hence, nVNS may lessen COVID-19-induced inflammation in symptomatic patients by focusing on the vagus nerve's auricular branch. The advantages of this technique are its low cost, non-invasive nature, and minimal adverse reactions [9].

Various devices are available for vagus nerve stimulation (VNS), including implantable devices and transcutaneous devices. nVNS works by delivering electrical impulses through the cervical or auricular branches of the vagus nerve to different brain regions, altering their signals and regulating brain physiology, chemistry, plasticity, and behavior. It’s worth noting that nVNS is a non-invasive method that has demonstrated effectiveness on par with the implantable device. The benefit of nVNS is that it has negligible side effects, such as minor skin irritation, and no adverse reactions [10]. Uehara et al. discovered that nVNS improved cognitive outcomes and inflammatory profiles in COVID-19 individuals [6]. On the other hand, other studies by Rangon et al. and Corrêa et al. showed that nVNS did not influence the clinical outcomes of COVID-19 infection [11,12]. Given the controversial and inconclusive data about the efficacy of this novel technique, specifically nVNS for COVID-19 infection, we performed this review to synthesize evidence on its effects on inflammatory biomarkers in COVID-19 through randomized controlled trials (RCTs) in the literature.

## Review

Methods

This study was conducted in line with the Preferred Reporting Items for Systematic Review and Meta-Analysis (PRISMA) [13]. This study protocol was registered at the International Prospective Register of Systematic Reviews (PROSPERO) (CRD42023449685).

Inclusion and Exclusion Criteria

We chose the following studies for this review: (1) study subjects: people who have been diagnosed with COVID-19; (2) nVNS as the experimental arm; (3) sham nVNS or standard of care as a means of comparison; (4) at least one biomarker was reported in the study, such as C-reactive protein (CRP), IL-6, IL-10, cortisol, fibrinogen, and D-dimer; and (5) RCTs. Case reports, case series, theses, conference abstracts, non-RCT studies, duplicated studies, studies not reported in English, and animal studies were all excluded from the list of articles.

Search Strategy

We searched five electronic databases: PubMed, Scopus, Web of Science, EMBASE, and Cochrane CENTRAL until July 2023 using relevant keywords. An example of the search strategy for PubMed was: (("Vagus Nerve"[MeSH] OR "Vagus Nerve" OR "transcutaneous vagus nerve stimulation" OR "tcVNS" OR "nVNS" OR "taVNS" OR "neuromodulation"[MeSH] OR "neuromodulation" OR "10th cranial nerve" OR "Cranial Nerve X") AND ("COVID-19"[MeSH] OR "COVID-19" OR "SARS-CoV-2"[MeSH] OR "SARS-CoV-2" OR "Coronavirus"[MeSH] OR "Coronavirus")).

Selection of Studies 

We conducted eligibility screening based on our inclusion criteria in two steps: first, we screened titles and abstracts for eligibility, and then we retrieved and screened full-text articles of the selected abstracts for inclusion in this review. Two reviewers independently conducted the literature search and screening. All conflicts were resolved through discussion with a third author. Additionally, we manually screened Google Scholar for relevant studies.

Data Extraction and Quality Assessment

We collected data from included studies using a standard data extraction template by a pair of distinct reviewers, and a third reviewer arbitrated any conflicts. The domains of the extracted data were: (1) the studies’ summary; (2) the population characteristics of each of the included studies; and (3) the studies’ results. Two reviewers independently assessed the risk of bias (RoB) in the included studies using the revised Cochrane RoB-2 tool for randomized trials. Each study was tagged as "low risk," "high risk," or "some concerns" [14].

Statistical Analysis

We conducted the statistical analysis using the RevMan 5.4 software (Cochrane, London, United Kingdom) for Windows 11. Each pooled effect size was represented as the mean difference (MD) and its 95% confidence interval (95% CI). We used the χ² test to assess heterogeneity between studies. Results of p < 0.1 were considered significant for heterogeneity, and a random effects model was used. I² tests were used to assess the degree of heterogeneity and were interpreted as follows: 0-40% might be important, 30-60% indicates moderate heterogeneity, 50-90% suggests substantial heterogeneity, and 75-100% reflects considerable heterogeneity. Otherwise, a fixed effects model was used to pool outcomes. Outcomes reported in at least two studies were included in the meta-analysis. Sensitivity analysis, by excluding one study at a time, was used to explore the source of heterogeneity in the analyses.

Results

**Figure 1:**
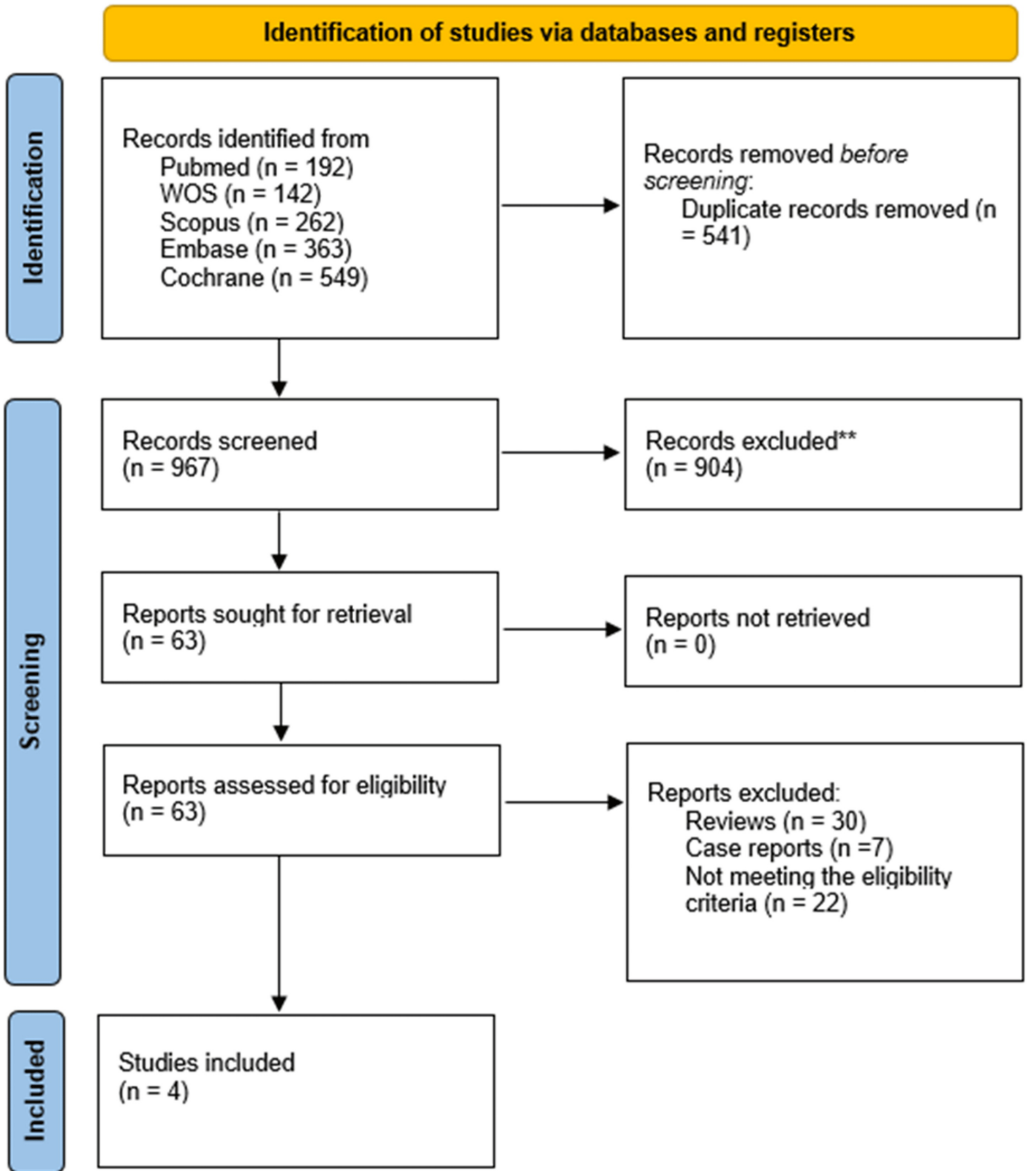
PRISMA flow chart Figure credit: [6,12,15,16]

Out of 967 papers initially retrieved for screening, 63 were eligible for full-text screening, and 59 were excluded for reasons detailed in Figure 1. Finally, our systematic review involved four studies (Figure 1) [6,12,15,16]. Two studies were excluded; although they assessed the use of nVNS, they had different outcomes that were not eligible for meta-analysis and were discussed in the discussion section [11,17].

Characteristics of the Included Studies

The four included RCTs [6,12,15,16] had a total of 180 patients diagnosed with COVID-19; 73 males were in the intervention group (intervention: active VNS), and 61 males were in the placebo group (normal hospital procedures). Uehara et al. [6] conducted a pilot RCT. The summary and baseline data of the studies are described in Tables 1-2, respectively.

**Table 1:** Characteristics of included studies RCT: Randomized controlled trial; CRP: C-reactive protein; IL-6: Interleukin-6; IL-10: Interleukin-10; TNF-α: Tumor necrosis factor-alpha; IL-1β: Interleukin-1 beta; HRV: Heart rate variability; PaO2/FiO2: Partial pressure of arterial oxygen to fraction of inspired oxygen ratio; CBC: Complete blood count; SAA: Serum amyloid A; DDIMER: D-dimer; CT: Cycle threshold (in PCR testing)

Study ID	Study design	Country	Number of centers	Total participants	Follow-up duration	Main inclusion criteria	Assessed outcomes
								Uehara et al. (2022) [6]	Pilot RCT	Brazil	1	21	7 days follow-up, 14 days follow-up	The patients should have a confirmed diagnosis of COVID-19, COVID-19, age over 18 years, be hospitalized and with symptom onset 1-10 days before admission, be conscious to consent to treatment, and answer questions about their clinical status.	The study primarily aimed to assess changes in inflammatory markers, including CRP, IL-6, IL-10, and cortisol levels.
Corrêa et al. (2022) [12]	RCT	Brazil	1	52	7 days follow-up, 14 days follow-up, 6 months follow-up	To be included, participants had to have symptoms of COVID-19 within ten days of the beginning of the first evaluation of this research.	Primary outcome: Inflammatory markers (IL-6, IL-10, cortisol, CRP). Secondary outcomes: Heart rate variability (HRV). COVID-19 symptom severity. Depression and anxiety levels. Attention and memory.
Tornero et al. (2022) [15]	RCT	Spain	1	97	NA	Patients should be ≥18 years old, have positive results for COVID-19 with cough and respiratory involvement, have oxygen saturation ≥92%, without the need for mechanical ventilation or severe respiratory insufficiency that will require immediate intubation, agree to use the nVNS device according to the instructions, and follow the requirements of the study, and be able to provide written informed consent.	Primary outcomes: Changes in inflammatory biomarkers, specifically CRP, procalcitonin, D-dimer, and cytokines (TNF-α, IL-6, IL-1β). Secondary outcomes: Respiratory function (PaO2/FiO2 ratio, oxygen saturation). Clinical events (ICU admission, need for ventilation, survival, discharge). Blood pressure (systolic and diastolic). CBC including absolute lymphocytes and white blood cell count. Other inflammatory markers (SAA, ferritin, haptoglobin).
Seitz et al. (2022) [16]	RCT	Austria	1	10	NA	Positive for SARS-CoV-2 by RT-PCR test (defined as a CT value less than 30), acute respiratory failure requiring non-invasive respiratory support, and PaO2/FiO2 <200.	Primary outcome: Reduction in inflammatory markers (e.g., CRP, TNF-alpha) over 7 days following aVNS treatment. Secondary outcomes: Reduction in coagulation markers (e.g., DDIMER). Increase in anti-inflammatory markers (e.g., IL-10).

**Table 2:** Baseline characteristics of included trials

Study ID		Age (years), mean (SD)	Sex, male n (%)	Diabetes n (%)	Hypertension n (%)	BMI kg/m^2^, mean (SD)	Smoke n (%)	Dyslipidemia n (%)	Asthma n (%)	Obesity n (%)	Duration of hospitalization/symptoms days mean (SD)
												Uehara et al. (2022) [6]	Case	53 (10.8)	7 (70%)	2 (20%)	7 (70%)	28.73 (3.1)	NA	NA	NA	4 (40%)	8 (3.3)
Control	44 (22.7)	4 (36.36%)	0 (0%)	4 (36.36%)	30.5 (5.4)	NA	NA	NA	6 (54.54%)	6 (3.2)		
Corrêa et al. (2022) [12]	Case	53 (17)	16 (61%)	7 (27%)	12 (46%)	30 (4)	1 (4%)	NA	NA	11 (42%)	9 (2)
	Control	57 (16)	10 (38%)	7 (27%)	15 (58%)	31 (6)	1 (4%)	NA	NA	13 (50%)	9 (2)
Tornero et al. (2022) [15]	Case	55.5 (13.9)	32 (68.1%)	10 (21.3%)	16 (34%)	NA	1 (2.1%)	22 (46.8%)	NA	NA	NA
	Control	61.3 (10.3)	38 (76%)	11 (22%)	25 (50%)	NA	1 (2%)	19 (38%)	NA	NA	NA
Seitz et al. (2022) [16]	Case	55.6 (8.69)	3 (60% )	2 (40%)	3 (60%)	35.32	1 (20%)	NA	0 (0%)	4 (80%)	10.4 (3.32)
	Control	53.2 (7.83)	2 (40%)	3 (60%)	2 (40%)	33.22	0 (0%)	NA	0 (0%)	4 (80%)	8.4 (2.06)

Quality Assessment of the Included Studies

**Figure 2:**
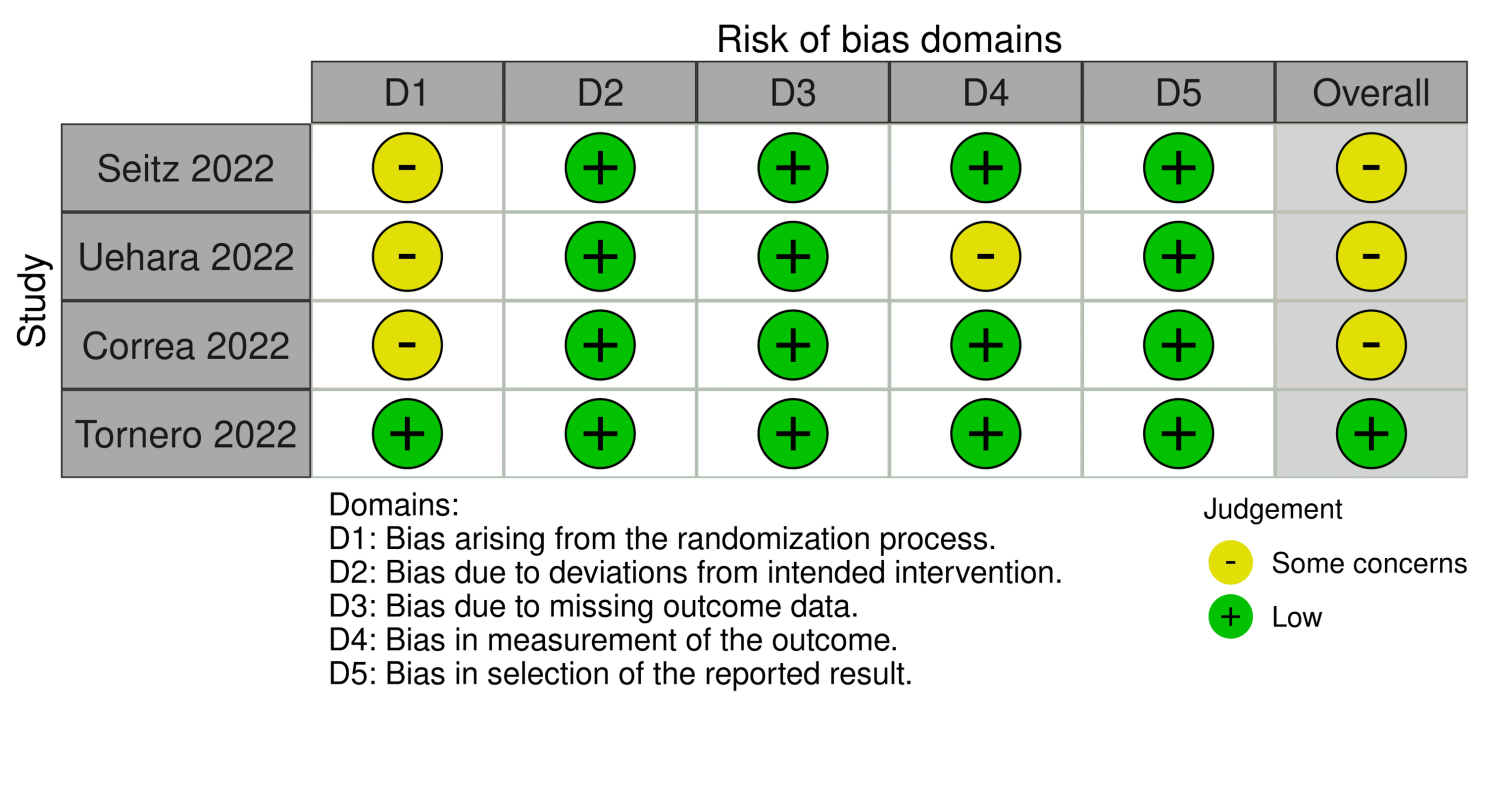
RoB-2 traffic light plot Image credit: [6,12,15,16] RoB: Risk of bias

**Figure 3:**
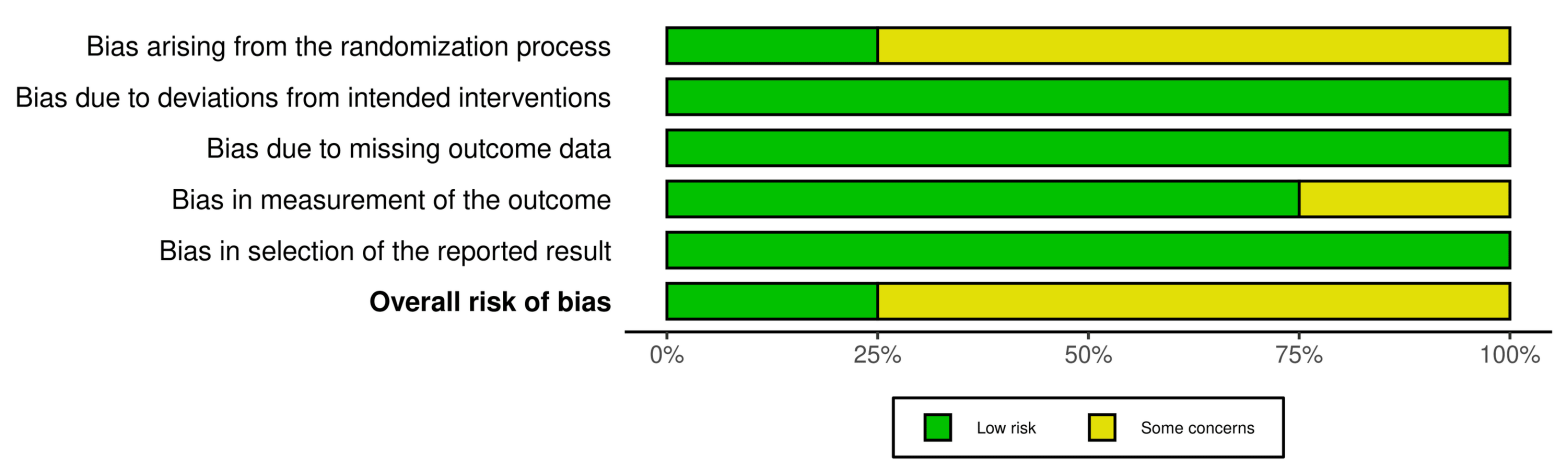
RoB2 summary plot Image credit: [6,12,15,16] RoB: Risk of bias

Three of the included RCTs showed some concerns about their RoB, while one had a low RoB according to ROB2. These studies had concerns in their randomization process, while one study had additional concerns regarding its measurement of the outcome (Figures 2-3).

*Meta-Analysis of CRP Levels*

**Figure 4:**
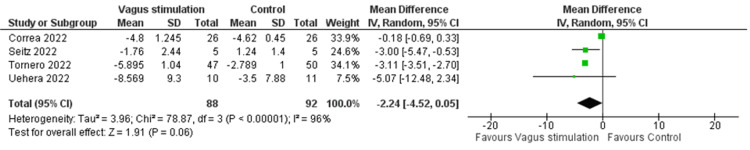
CRP levels in COVID-19 patients following nVNS Image credit: [6,12,15,16] CRP: C-reactive protein; nVNS: Non-invasive vagus nerve stimulation

**Figure 5:**
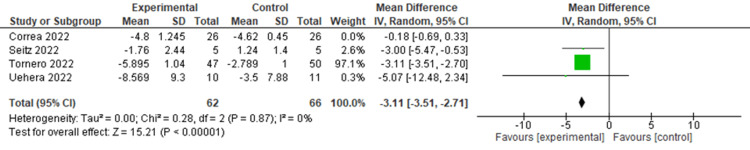
Sensitvitiy analysis of CRP levels in COVID-19 patients following nVNS Image credit: [6,12,15,16] CRP: C-reactive protein; nVNS: Non-invasive vagus nerve stimulation

The pooled analysis of four RCTs revealed a non-significant reduction in CRP levels following nVNS compared to control interventions, with an MD of -2.24 (95% CI: -4.52, 0.05; p = 0.06). The heterogeneity was significant (I² = 96%, p < 0.00001) (Figure 4). Heterogeneity was resolved after the exclusion of the Corrêa et al. study (I² = 0, p = 0.87), and the results became significant in favor of nVNS (MD = -3.11, 95% CI: -3.51, -2.27; p < 0.0001) (Figure 5).

Meta-Analysis of Cortisol Levels

**Figure 6:**
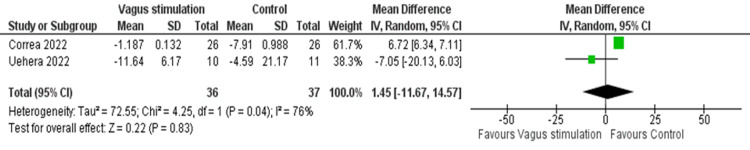
Pooled analysis of cortisol levels in COVID-19 patients after stimulation Image credit: [6,12]

Pooled data from two RCTs demonstrated a non-significant difference in cortisol levels in the intervention group compared to the control group (MD = 1.45, 95% CI: -11.67, 14.57; p = 0.83), with significant heterogeneity (I² = 76, p = 0.04) (Figure 6).

Meta-Analysis of IL-10 and IL-6 Levels

**Figure 7:**
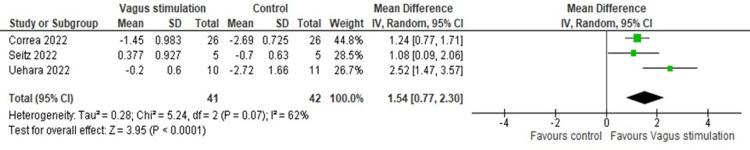
Pooled analysis of IL-10 levels in COVID-19 patients following nVNS Image credit: [6,12,16] IL: Interleukin; nVNS: Non-invasive vagus nerve stimulation

**Figure 8:**
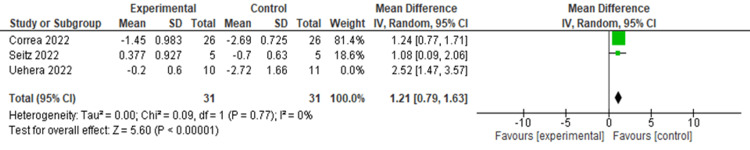
Sensitivity analysis of IL-10 level in COVID-19 patients after stimulation Image credit: [6,12,16] IL: Interleukin

Further, IL-10 levels were significantly increased after nVNS compared to control interventions (MD = 1.53, 95% CI: 0.77, 2.29; p < 0.0001), with significant heterogeneity (I² = 61.82, p = 0.073) (Figure 7). Heterogeneity was resolved after the exclusion of the Uehara et al. study from the analysis (I² = 0, p = 0.77), and results remained significant (MD = 1.21, 95% CI: 0.79, 1.63; p < 0.0001) (Figure 8).

**Figure 9:**
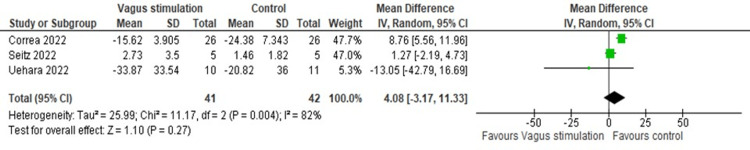
Pooled analysis of IL-6 levels in COVID-19 patients following nVNS Image credit: [6,12,16] IL: Interleukin; nVNS: Non-invasive vagus nerve stimulation

**Figure 10:**
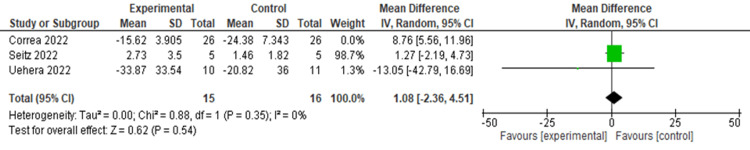
Sensitivity analysis of IL-6 levels of COVID-19 patients after stimulation Image credit: [6,12,16] IL: Interleukin

There was no significant difference in IL-6 levels after the nVNS intervention (MD = 4.07, 95% CI: -3.16, 11.32; p = 0.27), with significant heterogeneity (I² = 82.09, p = 0.004) (Figure 9). Heterogeneity was resolved after the exclusion of the Corrêa et al. study (I² = 0, p = 0.35), and the results remained insignificant (MD = 1.08, 95% CI: -2.36, 4.51; p = 0.540) (Figure 10).

Meta-Analysis of D-dimer Levels

**Figure 11:**
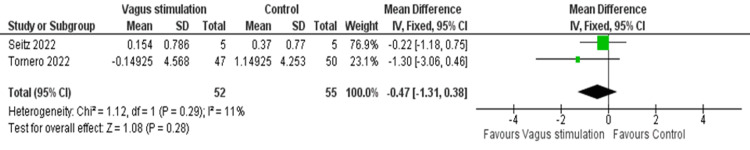
Pooled analysis of D-dimer levels in COVID-19 patients following nVNS Image credit: [15,16] nVNS: Non-invasive vagus nerve stimulation

The pooled analysis of two RCTs showed an insignificant change in D-dimer levels following nVNS treatment, and the studies were homogeneous (MD = -0.47, 95% CI: -1.31, 0.38; p = 0.28; I² = 11, p = 0.29) (Figure 11).

Discussion

This is the first systematic review aiming to assess the impact of nVNS on COVID-19-related inflammatory markers. Our analysis revealed that nVNS shows the potential to modulate inflammatory markers in COVID-19 patients. Specifically, there was a slight decrease in CRP and a nonsignificant reduction in cortisol levels. However, nVNS did not significantly affect IL-6 or D-dimer levels. Notably, there was a significant increase in IL-10, suggesting potential short-term immunomodulatory benefits. It is worth noting that all studies assessed the short-term effects of nVNS protocols (7-14 days).

Analysis of CRP, IL-10, and IL-6 revealed significant heterogeneity that was resolved through sensitivity analysis. The Corrêa et al. study was the main source of heterogeneity for the CRP and IL-6 analysis. Some of this heterogeneity may be explained by the varying number of sessions, with Corrêa et al. administering two sessions per day, while other studies implemented three or four sessions daily. The heterogeneity in the IL-10 analysis was mainly caused by the Uehara et al. study, which was a pilot study and had some concerns regarding its overall RoB. Additionally, some of the observed heterogeneity may be attributed to the use of different devices with varying frequencies, ranging from 1 Hz in some studies to 5 kHz in others.

The anti-inflammatory effects of nVNS are hypothesized to involve the CAP, reducing the release of pro-inflammatory cytokines, including IL-1, TNF-α, and IL-6 [18]. These cytokines play a key role in the altered immune response and cytokine storm observed in severe COVID-19 cases [19]. By reducing the production of these cytokines, nVNS could potentially improve clinical outcomes in COVID-19 patients by mitigating the severity of the inflammatory response, translating into a lower risk of severe complications [9]. These changes may be related to improvements in clinical outcomes, such as shorter hospital stays and a reduced need for mechanical ventilation, which could alleviate the burden on healthcare systems. The non-invasive nature of nVNS makes it an appealing option for patients who may not tolerate or who have contraindications to invasive treatments [20]. Furthermore, nVNS can be self-administered by patients or healthcare providers with minimal training, reducing the risk of transmission to healthcare workers [17].

Our primary analysis for CRP levels was insignificant. However, the sensitivity analysis revealed lower CRP levels in the nVNS group compared to the control group. Uehara et al. reported a significant reduction in plasma CRP levels (p = 0.04) in the active nVNS group after seven days of stimulation compared to the sham group, although other inflammatory markers and clinical manifestations showed no significant differences during the study period [6]. Additionally, Badran et al. reported some improvement in mental fatigue symptoms in a subset of patients who received vagal stimulation, while Rangon et al. did not observe significant improvement in their preliminary study [11,17]. The faster decrease in inflammatory biomarkers, compared to slower clinical improvement, suggests that a longer follow-up may be necessary in future studies [21].

IL-6 is a critical marker for monitoring COVID-19 patients, as elevated IL-6 levels are associated with poor prognosis, including ICU admission, ARDS, respiratory failure, multiple organ dysfunction, shock, and increased mortality risk [22]. Our analysis demonstrated a non-significant difference in IL-6 following nVNS. However, Corrêa et al. reported a significant reduction in IL-6 levels after nVNS treatment [12]. Similarly, Seitz et al. observed a decrease in IL-6 in the active VNS group, suggesting that nVNS may have a significant impact on COVID-19 outcomes by reducing pro-inflammatory cytokines [17,23]. The observed increase in IL-10 in our analysis after nVNS indicates that this intervention may support an anti-inflammatory environment. IL-10 is known to downregulate the inflammatory response, including the production of IL-6 and TNF-α, which are involved in the development of cytokine storms in severe COVID-19 cases [24]. The limited number of studies in each analysis should be taken into consideration to explain some of the heterogeneity and inconsistencies between different biomarkers.

The underlying mechanisms by which nVNS affects cortisol and IL-10 levels are likely multifactorial. While the exact pathways remain unclear, the vagus nerve-mediated activation of the CAP, involving acetylcholine release and inhibition of pro-inflammatory cytokines, may contribute to these effects [25,26]. Additionally, VNS modulates the autonomic nervous system by increasing parasympathetic activity and decreasing sympathetic activity, which can help reduce systemic inflammation by inhibiting the release of pro-inflammatory mediators. Regarding cortisol, our findings were not significant. Corrêa et al. reported no significant difference in cortisol levels after nVNS, while Uehara et al. observed a reduction in cortisol levels in the active group [6,12]. Cortisol plays a beneficial role in response to stress, limiting immune responses to prevent excessive inflammation when present in high concentrations [27].

Several studies in the literature evaluated clinical outcomes after nVNS. Rangon et al. reported no statistically significant difference between groups in clinical improvement [11]. They attributed this lack of difference to the advanced clinical deterioration of participants transferred from other hospitals, making them unsuitable for analysis. However, Badran et al. studied the use of self-administered nVNS in long-term COVID-19 patients experiencing mental health symptoms like anxiety and depression. Patients receiving the full four-week nVNS therapy showed significantly lower mental fatigue scores compared to those receiving sham treatment. While the small sample size limits generalizability, the study suggests potential benefits of at-home nVNS, improving compliance and easing mental symptoms [17]. These findings align with prior research on nVNS for depression treatment [28,29]. However, conflicting results emphasize the need for further research on the clinical utility of nVNS in COVID-19 patients. RCTs investigating optimal stimulation parameters are crucial for enhancing the evidence of their therapeutic psychological efficacy [6,12].

Strengths and limitations

To the best of our knowledge, we believe that this is the first systematic review on this topic, owing to the small amount of research published on such an approach in COVID-19. While the results are promising, it is important to note that the studies included in our review varied in their methodology in terms of sample size, follow-up duration, and nVNS protocol. The very small number of studies and sample sizes in this review, compared to other studies on COVID-19, may limit the generalizability of our results. However, this was necessary as we aimed to include only RCTs for the highest grade of evidence. Additionally, there were limitations to the quality of evidence available, with four studies showing a high RoB in the randomization process. We did not assess the clinical efficacy of nVNS in COVID-19 due to the limited number of studies and inconsistent measurements. Future larger studies with longer follow-up and correlation of clinical outcomes with inflammatory biomarkers are needed to understand the role of nVNS in COVID-19.

## Conclusions

This review presents preliminary data to support the potential use of nVNS as a treatment option for COVID-19 inflammation. Our results suggest that nVNS has an effect on inflammatory biomarkers in COVID-19, significantly increasing IL-10 levels. However, no significant effects of nVNS were found on CRP, IL-6, cortisol, or D-dimer levels. The findings of our review highlight the need for continued research into innovative treatments for COVID-19, and the potential of nVNS as a therapeutic adjunct warrants further investigation. Larger RCTs may be required before drawing firm conclusions on the efficacy of nVNS in COVID-19.
